# Genome analysis of yellow fever virus of the ongoing outbreak in Brazil reveals polymorphisms

**DOI:** 10.1590/0074-02760170134

**Published:** 2017-06

**Authors:** Myrna C Bonaldo, Mariela Martínez Gómez, Alexandre AC dos Santos, Filipe Vieira Santos de Abreu, Anielly Ferreira-de-Brito, Rafaella Moraes de Miranda, Marcia Gonçalves de Castro, Ricardo Lourenço-de-Oliveira

**Affiliations:** 1 Fundação Oswaldo Cruz-Fiocruz, Instituto Oswaldo Cruz, Laboratório de Biologia Molecular de Flavivírus, Rio de Janeiro, RJ, Brasil; 2 Fundação Oswaldo Cruz-Fiocruz, Instituto Oswaldo Cruz, Laboratório de Mosquitos Transmissores de Hematozoários, Rio de Janeiro, RJ, Brasil; 3 Instituto Federal do Norte de Minas Gerais, Montes Claros, MG, Brasil

**Keywords:** yellow fever virus, 2017 Brazil outbreak, amino acid changes

## Abstract

The current yellow fever outbreak in Brazil is the most severe one in the country in recent times. It has rapidly spread to areas where YF virus (YFV) activity has not been observed for more than 70 years and vaccine coverage is almost null. Here, we sequenced the whole YFV genome of two naturally infected howler-monkeys (*Alouatta clamitans*) obtained from the Municipality of Domingos Martins, state of Espírito Santo, Brazil. These two ongoing-outbreak genome sequences are identical. They clustered in the 1E sub-clade (South America genotype I) along with the Brazilian and Venezuelan strains recently characterised from infections in humans and non-human primates that have been described in the last 20 years. However, we detected eight unique amino acid changes in the viral proteins, including the structural capsid protein (one change), and the components of the viral replicase complex, the NS3 (two changes) and NS5 (five changes) proteins, that could impact the capacity of viral infection in vertebrate and/or invertebrate hosts and spreading of the ongoing outbreak.

Yellow fever virus (YFV) is the prototype member of the genus *Flavivirus* and family Flaviviridae. It is an arbovirus transmitted by the bite of infected mosquitoes in Africa and Americas, causing a disease with a large spectrum of symptoms, from mild disease to severe and deadly haemorrhagic fever in humans and New World non-human primates (NHP) (Vasconcelos & Monath 2016). Two main YFV cycles are described: the urban cycle involving the domestic mosquito *Aedes* (*Stegomyia*) *aegypti*, currently restricted to Africa, and the wild cycle in which humans are essentially infected by epizooties-affected NHPs, having sylvatic arboreal tree-hole breeding mosquitoes as vectors (species of *Aedes*, in Africa, and of *Haemagogus* and *Sabethes*, in the Americas). A rural or intermediate cycle may also occur in zones of emergence recorded in Africa (Monath & Vasconcelos 2015).

YFV is a single-stranded, positive-sense RNA virus with a genome of approximately 11 kb. Seven lineages have been identified: five in Africa (West Africa I and II, East Africa, East/Central Africa and Angola), and two in the Americas (South America I and II) (Bryant et al. 2007). Phylogenetic analysis provided evidence that the YFV circulating in the Americas is derived from a Western African lineage ancestor that emerged in Africa and was imported into the American East coast from West Africa during the slave trade (Vasconcelos et al. 2004, Bryant et al. 2007, Nunes et al. 2012).

The South American I is the most frequent genotype recorded in Brazil (Nunes et al. 2012, Monath & Vasconcelos 2015). Five lineages have been recognised in the South American genotype I, namely, 1A to 1E, which were associated with epidemics recorded during the cyclic expansions and retractions of YFV circulation in Brazil and other tropical American countries (Vasconcelos et al. 2004, d, de Souza et al. 2010). Since the turn of the century, the lineages 1D and 1E have been found in Brazil. However, since 2008, only YF viruses from lineage 1E have been detected in Brazil (de Souza et al. 2010, Nunes et al. 2012).

The most severe YFV epidemic recorded in Brazil in the recent decades has been reported since late 2016. Until the 10th epidemiological week of 2017, 1,558 cumulative cases with 137 confirmed YFV deaths were reported (COES 2017). Most importantly, this epidemic has rapidly and alarmingly spread eastward, reaching the most populated Brazilian regions where vaccine coverage is minor. Epizooties in NHPs and humans cases have been diagnosed in states considered YFV-free territories for almost 70 years.

Here, we present the complete genome sequence of two YFV samples collected during the current Brazilian epidemic along with a comparative analysis of recent YFV genome sequences characterised as belonging to the South American genotype I.

Blood samples were obtained from one recently dead and one dying howler-monkey (*Alouatta clamitans*) found on the Velho Rio farm (20º 17’ 08” S 40º 50’ 15” W), in Areinha, district of Ponto Alto, Municipality of Domingos Martins, state of Espírito Santo, Brazil, on February 20th and 22nd, 2017, respectively. Following centrifugation (2,000 *g* for 10 min), plasma samples were immediately frozen and transported to the laboratory in N_2_. Next, plasma samples were screened through reverse transcriptase polymerase chain reaction (RT-PCR), for which RNA was extracted from 140 μL of plasma using the QIAamp Viral RNA Mini Kit (Qiagen, Hilden, Germany), according to the manufacturer’s recommendations. RNA was eluted in 60 μL of AVE buffer and stored at -80ºC until use. Viral RNA was reverse transcribed using the High Capacity System (Applied Biosystems) with random hexamers according to the manufacturer’s recommendations. The reverse transcription reaction was carried out at 25ºC for 10 min, 37ºC for 120 min and 85ºC for 5 min. Further, the viral RNA was amplified by conventional PCR using PCR Master Mix (Promega), carried out at 95ºC for 2 min, followed by 30 cycles at 95ºC for 1 min, 58ºC for 1 min and 72ºC for 50 s, and then an extension at 72°C for 5 min. The set of primers utilised in this procedure were 5’-CTGTGTGCTAATTGAGGTGCATTG-3’ and 5’-ATGTCATCAGGCTCTTCTCT- 3’. The YFV infection of the monkeys was confirmed by the specific detection of a single amplicon with the expected YFV amplicon size of 650 bp (Fig. 1).

**Fig. 1:**
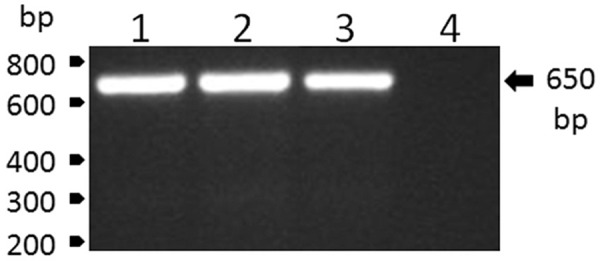
: detection of genomic RNA of yellow fever virus (YFV) by reverse transcriptase polymerase chain reaction (RT-PCR) analysis in plasma samples of howler monkeys from Velho Rio farm, in Areinha, Espírito Santo, Brazil. The numbered lanes refer to (1) positive control of the reaction that was performed with the YFV strain BeAn754036 obtained from insect C6/36 cell cultures; (2) YF RNA (strain ES-504/BRA/2017) and (3) YF RNA (strain ES-505/BRA/2017) that were extracted from howler-monkey plasma samples; and (4) a negative control of amplification. The size marker migration is indicated on the left of the figures, and the size of YFV amplicon is on the right.

To sequence of the full-length YFV genomes from the positive plasma monkey samples, 12 PCR amplicons were obtained (Supplementary data, Table). Viral RNA was reverse transcribed using the Superscript III First-Strand Synthesis System (Invitrogen) with random hexamers. Alternatively, we generated the first strand cDNA with the reverse primer P11R encoding the 3’UTR end (5´-AGTGGTTTTGTGTTTGTCA-3’) and further processed it with YF12F and YF12R for the synthesis of the second strand of cDNA. The cDNA was amplified by conventional PCR using GoTaq Green Master Mix (Promega) according to the manufacturer’s instructions. The thermocycling program in a Veriti 96-well thermocycler (Applied Biosystems) was used to amplify regions (1) to (11): 1 cycle at 95ºC for 5 min; 30 cycles at 95ºC for 40 s, at 50ºC for 40 s, and at 72ºC for 2 min; and finally 1 cycle at 72ºC for 10 min followed by incubation at 4ºC. For region (12), we applied 1 cycle at 95ºC for 5 min; 40 cycles at 70ºC for 40 s, 65ºC or 70ºC at 40 s, 72ºC at 50 s; and 1 cycle at 72ºC for 10 min and hold of 4ºC. Aliquots (3 µL of 50 µL) of amplified products were detected by electrophoresis on a 1% agarose gel, visualised by ethidium bromide staining and UV illumination, and purified with QIAquick PCR Purification Kit (QIAGEN). The amplicons were nucleotides that were directly sequenced without molecular cloning. Nucleotide sequencing reactions were performed using the ABI BigDye terminator V3.1 Ready Reaction Cycle Sequencing Mixture (Applied Biosystems) according to manufacturer’s recommendations. Nucleotide sequence was determined by capillary electrophoresis at the sequencing facility of Fiocruz-RJ (RPT01A - *Sequenciamento de DNA* - RJ). Raw sequence data were aligned and edited using the SeqMan module of LaserGene (DNASTAR Inc.).

The complete genome sequences of both YF viruses were deposited in the GenBank database under the following accession numbers: KY885000 for strain ES-504/BRA/2017 and KY885001 for strain ES-505/BRA/2017. When we compared these genomes, they displayed 100% identity. The evolutionary relationships of these two YFV strains from the ongoing outbreak with the modern YF sequences, primarily from South American genotype I, was established by phylogenetic analysis. Initially, we selected a set of sequences of the prM/E junction fragment using the Blast tool (https://blast.ncbi.nlm.nih.gov/Blast.cgi). The 666-bp sequence consists of the last 108 nucleotides of the prM gene, including the entire 225 nucleotides of the M gene, and the ﬁrst 333 nucleotides of the E gene. Nucleotide sequences were aligned using the CLUSTAL W program (Thompson et al. 1994) with selected YF viral sequences available at the GenBank database. A phylogenetic tree was generated by the Neighbour-joining method (Saitou & Nei 1987) using a matrix of genetic distances established under the Kimura-two parameter model (Kimura 1980), by means of the MEGA7 program (Kumar et al. 2016). The robustness of each node was assessed by bootstrap resampling (2,000 replicates) (Felsenstein 1985). The homologous region (prM/E) of a dengue virus strain available at the GenBank database (PaH881/88; Accession number: AF349753) was used as an outgroup. The Asibi prototype yellow fever strain (Accession number: AY640589) and the vaccine strain 17DD-Brazil (Accession number: DQ100292) were also incorporated into the analysis.

The South American YF sequences formed two major clusters: the South America I and the South America II genotypes, supported by 97% and 98% bootstrap values, respectively (Fig. 2). The South America genotype I clade is further divided into sub-clades as described by Vasconcelos et al. (2004) and de Souza et al. (2010). Sequence strains from ES-504/BRA/2017 (GenBank access number: KY885000) and ES-505/BRA/2017 (GenBank access number: KY885000) belonged to the South America genotype I, and grouped within the 1E sub-clade in conjunction with other modern strains detected in Brazil (years: 2002, 2004, 2008) and Venezuela (years: 1998, 2005-2007, 2010). The recent Brazilian and Venezuelan strains that were characterised from infections in humans and NHPs, also clustered in the 1E sub-clade (South America genotype I). Auguste et al. (2015) suggested that Brazil is the major source of YFV introduction into Venezuela. However, our data suggest that the most recent Brazilian YFV strains would have originated from a Venezuelan YFV strain, since the oldest strains in the E1 sub-clade were isolated in Venezuela in 1998 (Fig. 2). The acquisition in phylogenetic studies of additional complete YF genomes from ancestral and present circulating strains from humans, NHPs and mosquitos became necessary.

**Fig. 2:**
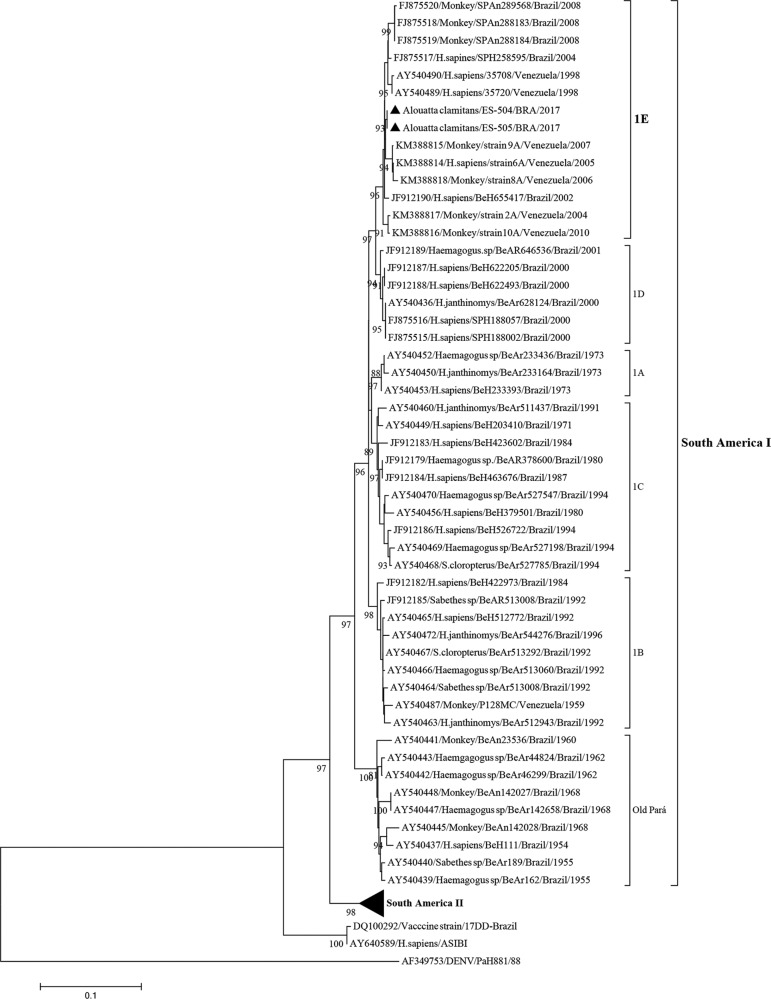
: phylogenetic analysis based on the prM/E junction region of yellow fever virus (YFV) strains analysed in the current study and 71 YFV sequences retrieved from the National Centre for Biotechnology Information (NCBI). Only bootstrap values up to 80% are shown. YFV genotypes are shown at the right side of the figure. The scale bar at the bottom represents 0.1 substitutions per nucleotide position (nt. subst./site). YFV first described in the current study are marked with a filled triangle. Accession numbers of the strains belonging to the South America genotype II are AY161929, AY161931-32, AY161934-35, AY161941-42, AY161944-45, AY161947-48, AY161950-51, AY540433-35, AY540446, and AY540457.

The comparison of the YFV precursor polyproteins obtained from complete genome sequences with those detected in Brazil and Venezuela since 1980 demonstrated eight unique and semi-conservative amino acid (aa) changes in the C, NS3 and NS5 proteins (Fig. 3). These changes map to the following polyprotein positions: (1) 108 for isoleucine (C protein); (2) 1572 for aspartic acid and 1605 for lysine (NS3 region); and (3) 2607 for arginine, 2644 for isoleucine, 2679 for serine, 3149 for alanine and 3215 for serine (NS5 protein). Interestingly, seven out of eight aa changes are located in the two important proteins of the viral replicase complex-NS3 and NS5 - and are perhaps associated with some selective advantage for viral fitness reflecting the ability of the virus to infect vertebrate and/or invertebrate hosts and spread the infection.

**Fig. 3:**
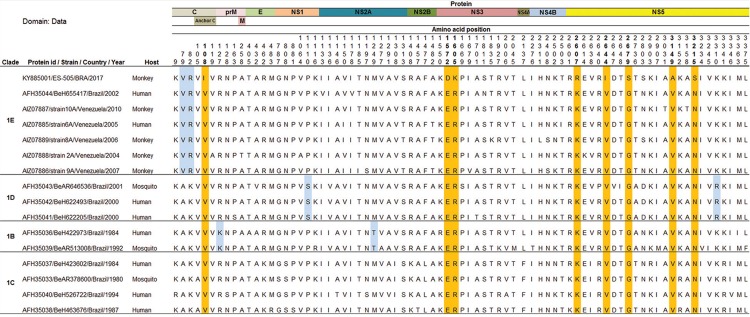
: amino acid (aa) differences revealed by the alignment of the precursor polyproteins of 16 Brazilian and Venezuelan yellow fever (YF) viruses detected since 1980. On the left of the alignment data, the identification of clades and yellow fever virus (YFV) sequences are supplied. On the top of the alignment, the YF viral proteins positions are indicated along with the aa positions of aa differences. The set of aa residues highlighted in blue indicate a related-clade pattern. The orange-highlighted aa indicate the position of the current YF sequences compared to the other YF sequences. For simplicity, only the ES-505/BRA/2017 strain sequence data were included in this figure.

However, it remains to be determined whether these specific aa changes are unique to the strains belonging to the ongoing outbreak. Alternatively, they, or at least some of them, could occur in some ancestral sequences that have not been sequenced so far. Hence, there are relatively very few complete YFV genomes from the Americas available at the GenBank database. On the other hand, this matter will be better clarified with the elucidation of the genomes of other circulating YF viruses in the current outbreak from infected mosquitos, NHPs and human biological samples. A wider understanding of the molecular epidemiology and evolution of YFV and their potential association with viral spreading and infectivity is of utmost relevance to determine the ancestral and modern YFV strains.
