# Supplementary material for: Genome analysis of yellow fever virus of the ongoing outbreak in Brazil reveals polymorphisms
Source: Mem Inst Oswaldo Cruz. 2017 Jun;112(6):447–51. doi: 10.1590/0074-02760170134 (PMC5446234; doi:10.1590/0074-02760170134)
Supplement: Supplementary file 1 [file 0074-0276-mioc-112-6-0447-suppl01.pdf]

TABLE

Primers used for amplification and sequencing of the whole genome of Brazilian yellow fever viruses described in the current study

Primer	Genome position	Sequence, 5'→3'	Amplicon size (bp)
YF1F	1-27	AGTAAATCCTGTGTGCTAATTGAGGTG	1400
YF1R	1380-1400	GCTGTGCCCTAATGACATACT	1400
YF2F	985-1006	AGGAATAACCGACAGGGATTTC	1339
YF2R	2304-2323	GCTCAAGCCACCAAACAATC	1339
YF3F	2144-2163	AAGGAAGGCAGCTCAATAGG	1444
YF3R	3566-3587	GTCCCTGTCTCTTCCTCAATAC	1444
YF4F	3426-3446	GGAGTGATGGTTGCTGGTATT	1449
YF4R	4853-4874	CTGCTATCAACTGAACCTCCTC	1449
YF5F	4506-4526	TCCACCCATTTGCACTCTTAC	586
YF5R	5070-5091	TTCACCTCAGTTTGGGATATGG	586
YF6F	4821-4842	CATGGAAGTTGGAGGGTAGATG	1358
YF6R	6156-6178	GACTCTCCTCTGGTCATCTCTTA	1358
YF7F	5840-5862	GTGGAGAGAGTGTTGGATTGTAG	916
YF7R	6736-6755	AAGAGATGTGGGTGGGTTTG	916
YF8F	6089-6108	GGGATGGTTGCTCCACTTTA	1440
YF8R	7507-7528	GAGGCTGGTATTTCCCTCTATG	1440
YF9F	7426-7445	CTTGGCCTCTGTTGCTATGT	1235
YF9R	8640-8660	TTCTCGTGACCTCCTCTATCC	1235
YF10F	8533-8554	CCCTTACAGGACTTGGCATTAT	1257
YF10R	9769-9789	TGAAAGTGGTGAGAGCAGAAG	1257
YF11F	9249-9268	GATGGGACACACGCATAACA	1441
YF11R	10670-10689	GGGCTGACATCCCACTATTT	1441
YF12F	10312-10340	GTACTCTGTGGATGCTGATCTGCAGCCCG	697
YF12R	10965-11008	AGTGGTTTTGTGTTTGTCATCCAAAGGTCTGCTTATTCTTGAGC	697
